# Do large thyroid nodules (≥4 cm) without suspicious cytology need surgery?

**DOI:** 10.3389/fendo.2023.1252503

**Published:** 2023-09-05

**Authors:** Seokmin Kang, Eunjin Kim, Sunmin Lee, Jin Kyong Kim, Cho Rok Lee, Sang-Wook Kang, Jandee Lee, Jong Ju Jeong, Kee-Hyun Nam, Woong Youn Chung

**Affiliations:** ^1^ Department of Surgery, College of Medicine, Severance Hospital, Yonsei University, Seoul, Republic of Korea; ^2^ Department of Surgery, Yongin Severance Hospital, Yongin-si, Republic of Korea

**Keywords:** large thyroid nodule, FNAB (fine needle aspiration biopsy), biopsy, huge nodule, 4 cm, follicular growth pattern, benign cytology, cancer incidence

## Abstract

**Background:**

Fine-needle aspiration biopsy (FNAB) is a good diagnostic tool for thyroid nodules; however, its high false-negative rate for giant nodules remains controversial. Many clinicians recommend surgical resection for nodules >4 cm owing to an increased risk of malignancy and an increased false-negative rate. This study aimed to examine the feasibility of this approach and investigate the incidence of malignancy in thyroid nodules >4 cm without suspicious cytology based on medical records in our center.

**Methods:**

This was a retrospective analysis of 453 patients that underwent preoperative FNAB for nodules measuring >4 cm between January 2017 and August 2022 at Severance Hospital, Seoul.

**Results:**

Among the 453 patients, 140 nodules were benign and 119 were indeterminate. Among 259 patients, the final pathology results were divided into benign (149) and cancerous (110) groups, and the prevalence of malignancy was 38.9% in the benign group and 55.5% in the indeterminate group. Among the malignancies, follicular carcinoma and follicular variants of papillary carcinoma were observed in 83% of the cytologically benign group and 62.8% of the indeterminate group.

**Conclusion:**

Preoperative FNAB had high false-negative rates and low diagnostic accuracy in patients with thyroid nodules >4 cm without suspicious cytologic features; therefore, diagnostic surgery may be considered a treatment option.

## 1 Introduction

Thyroid nodules are commonly observed, and clinically palpable huge nodules are observed in approximately 4–7% of the population (1, 2). Most thyroid nodules exhibit benign characteristics; however, the prevalence of malignant nodules has increased rapidly in recent years. Ultrasonography (US) is an effective test for thyroid nodules; many countries worldwide use objective imaging reporting systems to assess the risk of nodule formation, and fine needle aspiration biopsy (FNAB) is recommended. US-guided FNAB can be performed easily, has a high sensitivity and specificity of over 90%, and is the most reliable diagnostic tool for detecting thyroid nodular malignancies with a low false-negative rate and minimal complications (3, 4).

In 2007, using the Bethesda system, each nodule category was divided into stages from non-diagnosed to malignant (5). According to the Bethesda system, the expected incidence of malignancy in cytologically benign nodules ranges from 0% to 3% (6, 7). However, nodules larger than 4 cm, even with preoperative benign cytology, may increase the rate of false negatives and the risk of malignancy (8–12).

In a 2018 meta-analysis, although the rates of malignancy and false-negative FNAB results varied, the differences were not greater in larger nodules in most studies. There is no need for immediate surgical resection when a large thyroid nodule is found to be cytologically benign (13). Although many studies have shown no difference between small and large nodules concerning false negatives of FNAB, many clinicians recommend surgical resection for nodules larger than 4 cm due to an increased risk of malignancy and an increased false-negative rate, even when the FNAB result is benign (14–16).

According to the 2015 American thyroid association management (ATA) guidelines, no further diagnosis or treatment is required for cytologically benign nodules. However, the guidelines specify “uncertainty” as to whether the risk of malignancy is higher than that of small nodules, even if the thyroid nodule is larger than 4 cm with benign cytology, and whether it should be managed differently from small nodules (17). Inconsistent clinical guidelines for managing large nodules can lead to unnecessary surgery and costs and increased patient morbidity.

Therefore, this study aimed to examine the feasibility of this approach by investigating the incidence of malignancy in thyroid nodules larger than 4 cm with benign cytology, based on medical records at our center.

## 2 Materials and methods

### 2.1 Patients and methods

We reviewed consecutive patients referred to Severance Hospital between January 2017 and August 2022. We retrospectively collected data for all nodules larger or equal to 4 cm that showed benign or indeterminate cytological results on FNAB.

**Figure 1:**
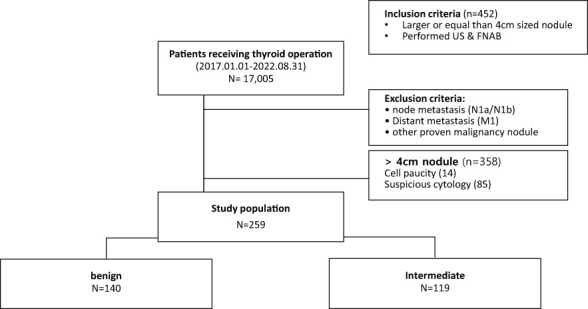
Flowchart of selection of patients with thyroid nodules larger than or equal to 4 cm without suspicious cytology.

A total of 17,005 patients underwent thyroid surgery during this period, and 452 had large nodules measuring over 4 cm on preoperative US. Exactly 358 cases were confirmed, except for cases involving distant metastasis, lymph node metastasis, and suspected lesions in other thyroid gland nodules. Among them, 259 cases were confirmed to have undergone FNAB before surgery, except for 99 cases with category 1 and category 4 or higher. Finally, FNAB helped identify 140 benign and 119 indeterminate cases (**Figure 1**) (18).

### 2.2 Diagnosis and evaluation

All patients underwent thyroid ultrasonography (US)-guided FNAB and serum thyroid-stimulating hormone (TSH) measurement. The thyroid US-guided FNAB procedure and subsequent evaluation of FNAB cytology slides were performed and evaluated by a radiological specialist and a cytopathologist, respectively, at our hospital. The FNAB results and final pathology reports included in the study were classified based on criteria and terminology analogous to those of the Bethesda System for Reporting Thyroid Cytopathology. Patients with a benign nodule larger than or equal to 4 cm underwent thyroid lobectomy and isthmusectomy. Indication of thyroidectomy completion was based on the presence of malignancy within the resected specimen.

### 2.3 Statistical analysis

Statistical analysis was performed using the Statistical Package for Social Sciences (SPSS) version 26.0 for Windows (IBM Corp., Armonk, NY, USA). Data were expressed as counts and percentages, means ± standard deviation (SD), or medians and ranges. Student’s t-test, chi-squared test, and Fisher’s exact test were used as appropriate. Statistical significance was set at p <0.05. The study protocol was approved by the Institutional Review Board of the Yonsei University College of Medicine, because of the retrospective nature of the study.

## 3 Results

The 259 study participants comprised 55 men and 204 women (ratio 1:3.71), with a mean age of 45.6 ± 15.1 years. Almost all participants had a palpable neck mass, and 28.6% complained of symptoms such as pain, breathing discomfort, and difficulty in breathing. The overall primary tumor malignancy rate was 45.9% (N = 119). The mean primary tumor size was 5.20 ± 0.93 cm on US and 5.02 ± 0.93 cm on final pathology. Each nodule was benign in 229 (88.4%) cases, indeterminate in 26 (10.0%), and suspicious in 4 (1.5%) on US. Of the 259 operations, 245 were classified as less than total thyroidectomies (94.6%) and 14 as total thyroidectomies (5.4%, including four cases of Graves’ disease with multinodular goiter, two of both whole glands involving huge nodules, five of multinodular goiter, and three after confirmation using the cryosection test during surgery) (**Table 1**).

**Table 1:** Demographic and pathologic features of 259 patients with thyroid nodules larger than or equal to 4 cm without suspicious cytology.

		N = 259
Age (years), mean ± SD		45.6 ± 15.1 (11-87)
Sex (Male:female) ratio, n (%)		55: 204 (21.2%:78.8%)
Thyroid cancer Family Hx		29 (11.2%)
Symptom (dyspnea, discomfort, pain)		74 (28.6%)
Benign/cancer, n(%)		140: 119 (54.1%: 45.9%)
Size(mm),	USG	51.9 ± 9.28 (40–110)
mean ± SD(range)	Pathology	50.2 ± 9.28 (40-100)
FNAB	Benign	149 (57.5%)
	Indeterminate	110 (42.5%)
USG feature		
Benign		229 (88.4%)
Indeterminate		26 (10.0%)
Suspicious		4 (1.5%)
Operation characteristics, n (%)		
Lobectomy/+subtotal/total		213/32/14 (86.9%/12.4%/5.4%)
Less total/Total		245: 14 (94.6%: 5.4%)

These results were divided into 149 cytologically benign and 110 indeterminate groups based on FNAB (**Table 2**). There were no significant differences in sex, age, family history, symptoms, surgical extent, and size of US and pathology reports, except for US features (p <0.001). The prevalence of malignancy was 38.9% in the benign group and 55.5% in the indeterminate group, and it sequentially increased in the more suspicious group. However, the difference was not significant.

**Table 2:** Demographic and pathologic features of 149 patients with benign cytology and 110 patients with indeterminate cytology of thyroid nodules larger than or equal to 4 cm.

Variable	Benign cytology (n=149)	Indeterminate cytology (n=110)	P-value
Age (years), mean ± SD	46.46 ± 14.65 (14- 81)	44.55 ± 15.79 (11- 87)	0.48
Sex (male: female) ratio, n (%)	28: 121 (18.8%:81.2%)	27: 83 (24.5%: 75.5%)	0.28
Thyroid cancer FHx	14 (9.4%)	15 (13.6%)	0.28
Symptom	48 (32.2%)	26 (23.6%)	0.13
Benign/cancer, n(%)	91: 58 (61.1%: 38.9%):	49: 61 (44.5%: 55.5%)	0.10
Size (mm), mean ± SD (range)			
USG	52.89 ± 9.303 (40 – 100)	50.73 ± 9.62 (40 – 85)	0.46
Pathology	50.64 ± 8.86 (40 - 82)	49.70 ± 9.83 (40 - 100)	0.60
USG feature			<0.001
Benign	145 (97.3%)	84 (76.4%)	
Indeterminate	3 (2.0%)	23 (20.9%)	
Suspicious	1 (0.7%)	3 (2.7%)	
Operation characteristics, n (%)			0.38
Less total/Total	141: 8 (94.6%: 5.4%)	104: 6 (94.5%: 5.5%)	

Among the 259 cases, 140 (54.1%) were diagnosed with benign lesions. Of the 140 benign lesions, adenomatous hyperplasia was present in 65 patients (45.9%), while follicular adenoma was found in 54 (38.6%). Of the 119 patients with histologically confirmed thyroid cancer, 75 had papillary carcinoma (63.0%). Moreover, 22 follicular carcinomas (18.5%), 5 oncocytic carcinomas (4.2%), and 7 poorly differentiated carcinomas (5.9%) were confirmed. The patients were divided into benign (149) and indeterminate (110) cytology groups. In both groups (benign and indeterminate), benign lesions appeared as adenomatous hyperplasia and follicular adenomas (**Table 3**).

**Table 3:** Final histologic classification in the pathology report.

	Total (n=259)	Benign (n=149)	indeterminate (n=110)
Benign, n (%)	140 (54.1%/100%)	91 (61.1%/100%)	49 (44.5%/100%)
Adenomatous hyperplasia	65 (25.1%/46.4%)	48 (32.2%/52.8%)	17 (15.4%/34.7%)
Follicular adenoma	54 (20.8%/38.6%)	33 (22.1%/36.3%)	21 (7.4%/15.1%)
Oncocytic cell adenoma	20 (7.4%/19.3%)	9 (6.0%/9.9%)	11 (10.0%/22.5%)
Lymphocytic thyroiditis	1 (0.3%/0.7%)	1 (0.7%/1.1%)	0 (0%)
Malignancy, n (%)	119 (45.9%/100%)	58 (38.9%/100%)	61 (55.5%/100%)
NIFTP	10 (3.9%/8.4%)	5 (3.4%/8.6%)	5 (4.5%/8.2%)
PTC	75 (29.0%/63.0%)	36 (24.2%/62.1%)	39 (35.5%/63.9%)
Follicular	22 (8.5%/18.5%)	11 (7.4%/19.0%)	11 (10.0%/18.0)
Oncocytic	5 (1.9%/4.2%)	1 (0.7%/1.7%)	4 (3.6%/6.6%)
Poorly differentiated	7 (2.7%/5.9%)	5 (3.4%/8.6%)	2 (1.8%/3.3%)

In the benign cytology group, the malignancy rate was 38.9%: 5 non-invasive follicular thyroid neoplasm with papillary like nuclear features (NIFTP) (8.6%), 36 papillary carcinomas (62.1%), 5 conventional papillary thyroid cancers (PTCs) (8.6%), 30 follicular variants of PTCs (51.7%), and 1 oncocytic variant of PTCs (1.7%). Other histologic types diagnosed as malignancy included 11 follicular carcinomas (19.0%), 1 oncocytic carcinoma (1.7%), and 5 poorly differentiated carcinomas (8.6%), of which four cases were accompanied by follicular carcinomas.

In the indeterminate cytology group, the malignancy rates were 55.4%: 5 NIFTP (8.2%), 39 papillary carcinomas (63.9%), 5 conventional papillary thyroid cancers (PTCs) (8.2%), 31 follicular variants of PTCs (50.8%), and 3 solid variants of PTCs (4.9%). Other malignancies were one follicular carcinoma (1.6%), four oncocytic carcinomas (6.6%), and two poorly differentiated carcinoma (3.3%), of which one case was associated with anaplastic carcinoma (**Table 4**).

**Table 4:** Comparison of the detailed PTC subtypes and malignant groups in the pathological reports.

Malignancy		Benign (n=58/38.9%)	indeterminate (n= 61/55.5%)
NIFTP		5 (3.4%)	5 (4.5%)
PTC variant	Classic	5 (3.4%)	5 (4.5%)
	Follicular	29 (19.5%)	30 (27.3%)
	Macro-follicular	1 (0.7%)	1 (0.9%)
	Oncocytic	1 (0.7%)	–
	Solid	–	3 (2.7%)
Follicular carcinoma		11 (7.4%)	1 (10.0%)
Oncocytic carcinoma		1 (0.7%)	4 (3.6%)
Poorly differentiated		5(3.4%) * 4 cases associated with Follicular carcinoma	2 (1.8%) * 1 case associated FVPTC * 1 case associated with anaplastic carcinoma

*n, Number; NIFTP, Noninvasive follicular thyroid neoplasm with papillary-like nuclear features; PTC, Papillary thyroid carcinoma; FVPTC, Follicular variant of papillary thyroid carcinoma.

Among the 259 patients, the final pathology results were divided into 149 benign and 110 cancer groups. There were no significant differences in sex, age, family history, symptoms, size, or preoperative ultrasound findings.

The prevalence of malignancy was 38.9% and 55.5% in the benign and indeterminate groups, respectively. Among the malignancies, follicular carcinoma and follicular variants of papillary carcinoma were observed in 83% of the cytologically benign group and 62.8% of the indeterminate group.

## 4 Discussion

The consideration of tumor size as a risk factor for thyroid malignancy remains controversial (19–21). While US-guided FNAB is commonly used as a preoperative diagnostic tool because of its high accuracy (12, 17, 22, 23), there have been reports of high malignancy rates and false negatives associated with large thyroid nodules.^6-10^ Several studies have reported that increased tumor size is not an independent factor predicting malignancy, while many others have concluded that a larger tumor size leads to an increased risk of malignancy (12, 13, 19). According to these conclusions, thyroid nodules larger than 4 cm without suspicious cytology findings before surgery increase the risk of being diagnosed with a malignancy in the final pathology report. The 2015 ATA guidelines suggest a near-total or total thyroidectomy as the initial surgical procedure for patients with thyroid tumors larger than 4 cm.^15^ Therefore, in patients in whom benign nodules are expected, if malignant nodules are observed, completion of thyroidectomy and central compartment lymph node dissection may be required (24, 25), which causes inconvenience to both the doctors and the patients, besides the emotional and the financial burdens on the families.

Although US-FNAB has demonstrated excellent diagnostic performance, it has several limitations in diagnosing large thyroid nodules. First, larger nodules are often heterogeneous and contain areas of both benign and malignant tissues. Therefore, a small sample obtained using FNAB may miss the malignant portion of the nodule, leading to false-negative results (26, 27). Second, larger nodules may have a more complex architecture, making it difficult for pathologists to correctly interpret biopsy samples. This can lead to false-negative results due to incorrect interpretation of the biopsy sample (28). Third, large nodules may have a higher proportion of cystic areas, which may not yield adequate tissue samples for analysis. Cystic areas may also contain fluid that dilutes the cellular material obtained by FNAB, thereby reducing biopsy accuracy (26, 27). Finally, the position of the nodule within the thyroid gland can affect biopsy accuracy. Nodules located deep within the gland may be more difficult to access with a needle, making it more challenging to obtain an adequate sample (26–28).

Several recent studies indicate that the prevalence of malignancy is higher in larger thyroid nodules; further, the diagnostic factors affecting the diagnosis of follicular tumors, such as reliance on the skill of the operator/interpretation cytologist and false-negative results due to incorrect sampling from small nodules, lead to high false-negative results ranging from 13–50%. In this study, the sensitivity of the combination of FNAB and core needle biopsy(CNB) for nodules larger than 4 cm was 78.3, the specificity was 59.8, the positive predictive value was 77.9, the negative predictive value was 60.4, and the accuracy was 71.8.

In this study, 63 patients underwent CNB. Of these, 31 patients had it as their first test and 32 had it as an additional test after FNAB. Of the 32 patients, 9 were found to be category 3 on FNAB, with 2 in category 2, 6 in category 3, and 9 in category 4 after CNB. There were 5 category 1s on FNAB, with 3 category 4s and 2 category 2s after CNB. 10 patients were category 2 on FNAB, of which 8 were category 4 after CNB and 2 were category 3. Diagnostic surgery is often recommended for nodules larger than 4 cm at this institution, and patients who have been examined and followed up at other hospitals for many years have visited this hospital for surgery, so there were few cases where CNB was performed after FNAB. It seems that it will be difficult to mention the accuracy of CNB with a small sample size, which is one of the limitations of this study.

In 2022, the Department of Radiology at our institution conducted a study to compare the diagnostic outcomes of US-guided FNAB and CNB for the same nodule using surgical specimens. In a study of 89 thyroid nodules larger than 20 mm, Hyuk Kwon et al. argued that “Both FNA and CNB had significantly higher rates of AUS/FLUS (63.1% vs. 21.7%) or indeterminate results (78.9% vs. 54.3%) in malignant nodules compared to benign nodules, CNB showed a potential to provide improved diagnostic sensitivity for thyroid cancer, especially when a conclusive diagnosis is limited with FNA. Also, regarding nodule size, they found no significant difference in definitive and inconclusive rates between FNA and CNB for nodules in the 20- to 40-mm size range. However, for nodules larger than 40 mm, the inconclusive rate was significantly higher in malignant nodules with FNA, while there was no significant difference between malignant and benign nodules with CNB.” (12, 29, 30).

FNAB is the most useful test when the cytologic diagnosis is classic PTC; however, it has limitations in the diagnosis of follicular or oncocytic neoplasms and follicular variants of PTCs (24, 25, 28). In this study, except for classic PTCs and NIFTP, follicular carcinoma and follicular variants of PTCs were found in approximately 82.7% of the cytologically benign group and 62.3% of the indeterminate group. FNAB had a high false-negative rate in patients with thyroid nodules larger than 4 cm. These nodules exhibit the same follicular growth patterns found in follicular thyroid carcinoma, oncocytic carcinoma, and follicular variants of PTCs (31).

In this study, the number of FNABs was not analyzed. However, due to the nature of the institution, which is a tertiary medical institution, many patients had been followed up for as long as a decade; therefore, many cases showed repeat benign FNAB findings for several years but were malignant after surgery. In addition, many patients with nodules larger or equal to 4 cm underwent surgery. Because our institution recommends diagnostic surgery for nodules larger or equal to 4 cm, data regarding many cases could be collected, even though it was a single institution.

In this study, 58 out of 149 patients (38.9%) who were initially classified as benign on preoperative FNAB and subsequently underwent surgery were found to have malignant nodules. Similarly, 61 out of 110 patients (55.5%) who were classified as indeterminate and underwent surgery were found to have malignancy. It should be noted that due to the characteristics of our center, priority surgery was considered for patients with long-term follow-up of giant nodules and the size of the thyroid nodule continued to increase, as well as cases where it was challenging to definitively rule out malignancy based on imaging studies. These factors may have introduced a selection bias that increased the overall risk of malignancy. Due to concerns regarding overdiagnosis and overoperation of the thyroid gland worldwide, diagnostic surgery for cytologically benign and indeterminate nodules should only be considered when it is challenging to definitively rule out malignancy clinically or when there are observable imaging changes.

Furthermore, molecular analysis using the following markers (BRAF V600E, NRAS, HRAS, KRAS, RET/PTC, and PAX8/PPARg) to evaluate preoperative genetic mutations and rearrangements has been shown to have significant diagnostic value in thyroid nodules with indeterminate cytology (32). In this study, the need for surgery could have been considered by adding molecular tests to the FNAB results; however, we did not compare them because consistent molecular tests were not performed in the cytologic indeterminate group (33, 34). However, in the pathology report, 82.7% of cases in the benign group and 62.3% in the indeterminate group showed malignant findings with a follicular growth pattern; therefore, it would have been difficult to expect meaningful results from molecular tests, such as BRAF mutation.

Several studies have shown that molecular tests cannot explain pathological findings in tumors larger or equal to 4 cm in size, but are useful in tumors smaller than 4 cm (34). However, molecular studies have not yet been widely adopted owing to their low cost-effectiveness. Further research is needed to explore alternative diagnostic tools for assessing benign thyroid nodules larger or equal to 4 cm in size.

Additionally, intraoperative frozen sections may be considered an option for determining the extent of surgical resection. However, the accuracy is low for follicular variants of PTCs and follicular thyroid carcinoma (35, 36). As shown in this study, thyroid nodules larger or equal to 4 cm tended to show malignant histopathology of follicular neoplasms, such as follicular carcinoma and follicular variants of PTCs. Therefore, the use of frozen sections is not recommended during surgery.

It was not possible to analyze the differential malignancy rate of FNAC diagnosed as AUS/FLUS according to nuclear or structural atypia in an indeterminate group and to compare clinicopathologic characteristics in this study. This study was conducted retrospectively, and the detailed specification of nuclear or structural atypia in the pathology results could not be confirmed in this study, which may be one of the limitations of this study (37).

## 5 Conclusions

Preoperative FNAB had high false-negative rates in patients with benign cytology and thyroid nodules measuring ≥ 4 cm, particularly in cases presenting growth patterns resembling those found in FTC, oncocytic carcinoma, and PTC follicular variants. These neoplasms are difficult to diagnose using preoperative FNAB. Therefore, for such findings, clinicians should consider performing a diagnostic lobectomy as a treatment option.

## Data availability statement

The original contributions presented in the study are included in the article/supplementary material. Further inquiries can be directed to the corresponding author.

## Ethics statement

The studies involving humans were approved by Institutional Review Board of the Yonsei University College of Medicine. The studies were conducted in accordance with the local legislation and institutional requirements. The participants provided their written informed consent to participate in this study. Written informed consent was obtained from the individual(s) for the publication of any potentially identifiable images or data included in this article.

## Author contributions

SK and S-WK were responsible for conception and design. SK wrote the manuscript and carried out review and revision of the manuscript. All authors contributed to the article and approved the submitted version.
